# Ocular loaiasis in France: the first case report from Brittany

**DOI:** 10.1186/s12348-024-00437-7

**Published:** 2024-11-06

**Authors:** Sarah Kerkouri, Thomas Monfort, Dorothée Quinio, Béatrice Cochener-Lamard

**Affiliations:** 1 Univ Brest, CHU Brest, Brest, F-29200 France; 2 Service d’ophtalmologie, CHU de Brest, Brest, F-29200 France; 3 INSERM, UMR 1101, Brest, F-29200 France; 4 Université Paris-Saclay, Gif-sur-Yvette, France; 5 Laboratoire de Parasitologie et Mycologie, CHU Brest, Brest, F-29200 France

**Keywords:** Loaiasis, Subconjunctival infection

## Abstract

We report the case of a 21-year-old Cameroonian woman residing in France for one year, who presented to our department with left eye discomfort and itching. Examination revealed a mobile translucent cord beneath the nasal-inferior conjunctiva, prompting suspicion of loaiasis. Anesthesia was administered for extraction, revealing a 31 mm male Loa loa. A positive microfilarial load, albeit low, confirmed the diagnosis and the diagnostic workup excluded other locations. Treatment with diethylcarbamazine was well-tolerated. This case highlights the importance of considering loaiasis in non-endemic regions and underscores the need for interdisciplinary collaboration in its diagnosis and management.

**Supplementary Information:**

The online version contains supplementary material available at 10.1186/s12348-024-00437-7.

Loaiasis, or African eye worm, is a chronic parasitic infection affecting approximately 20 million individuals [1] worldwide in endemic areas, mainly west and central Africa, with imported cases reported elsewhere. In Paris and its suburbs, 167 imported cases were described between 1993 and 2013 [2]. Cameroon was the main country of exposure, and the eyeworm was reported in 23% of patients. Loaiasis is transmitted by a *Chrysops* fly that inoculates the *Loa loa* larvae through its bite. We report the case of a 21-year-old Cameroonian woman who had been a student and resident in France for one year, with no travel history to endemic regions since her arrival. In September 2023, she presented to the Ophthalmic Emergency Department of Brest University Hospital with discomfort and itching in the left eye. The visual acuity measured with the Monoyer scale was 10/10 in the right eye and 7/10 in the left eye. Examination revealed a mobile translucent cord beneath the nasal-inferior conjunctiva of the left eye (Fig. 1 and the video in the supplementary file). The anterior chamber was quiet, the fundus was normal and the right eye examination was unremarkable. In this case, due to the patient’s anxiety and discomfort, a short general anesthesia was administered for the procedure. One living nematode was extracted. Parasitological examination identified a male *Loa loa* of 31 mm length (Fig. 2). While the patient had no eosinophilia, the serological test using ELISA method (test NovaLisa Filariasis, NovaTec) was negative, with a positive but low microfilarial load (< 0.05/µL). No other *Loa loa* locations were identified. Following a three-week wait for medication delivery, the patient was hospitalized in the Infectious Diseases Department to monitor diethylcarbamazine treatment tolerance. The treatment began with 1/32 tablets of 100 mg, progressively doubling to an effective dose of 200 mg twice daily from day 8. The total duration of diethylcarbamazine treatment was 28 days. The treatment was well tolerated, with mild headaches managed with acetaminophen. Ophthalmic follow-up was uneventful. This case underscores the need to consider loaiasis in at-risk individuals in non-endemic areas, highlighting the need for interdisciplinary collaboration with microbiologists and infectiologists and reconsideration of treatment availability in non-endemic areas. Loaiasis, currently not on the WHO list of neglected tropical diseases, warrants attention, as a recent study showed a 14.5% attributable mortality risk [3], which could change owing to the MorLo projects.

**Fig. 1:**
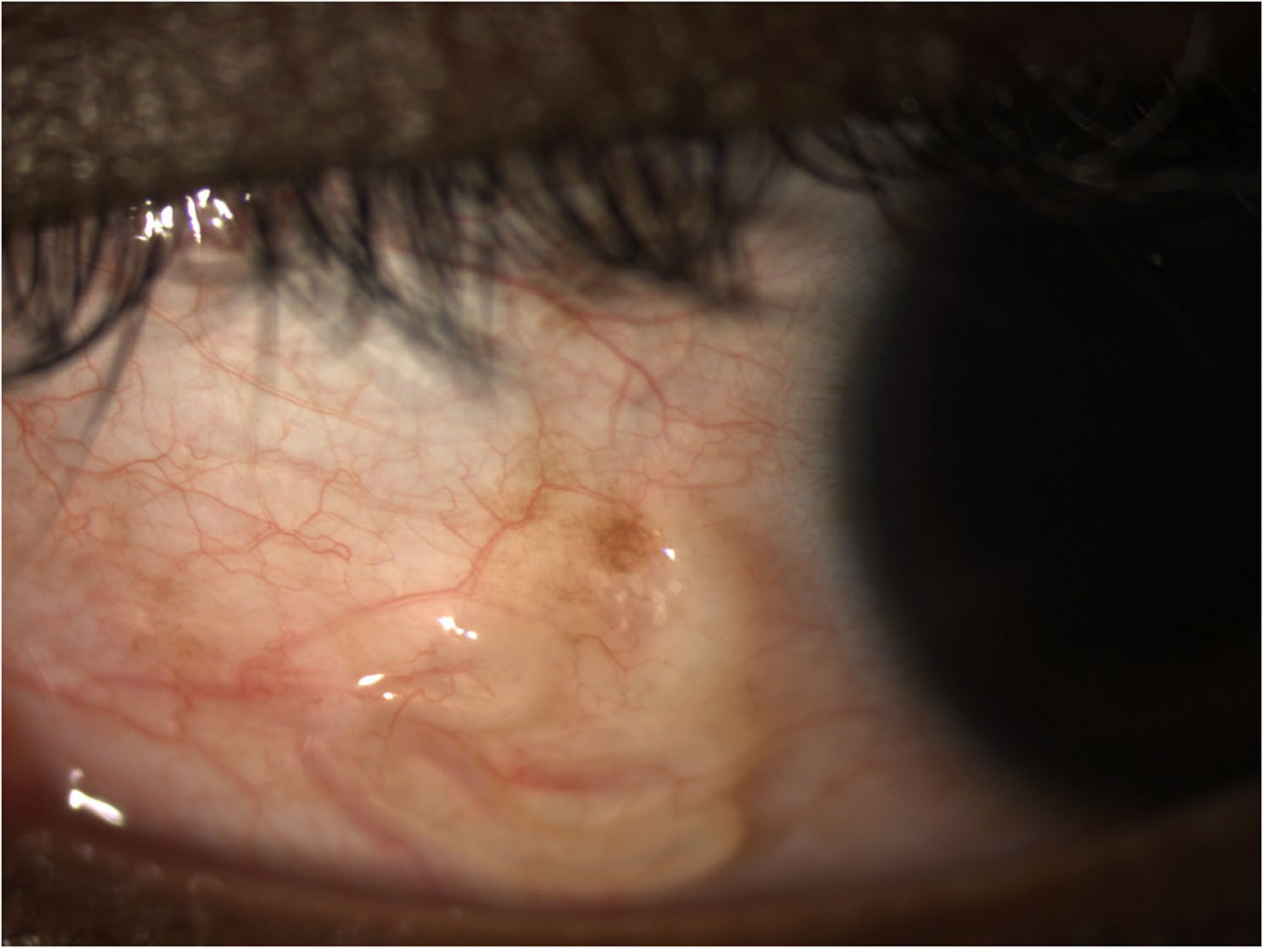
Slit lamp examination of the left eye revealed a translucent cord beneath the nasal-inferior conjunctiva, suggestive of a diagnosis of loaiasis

**Fig. 2:**
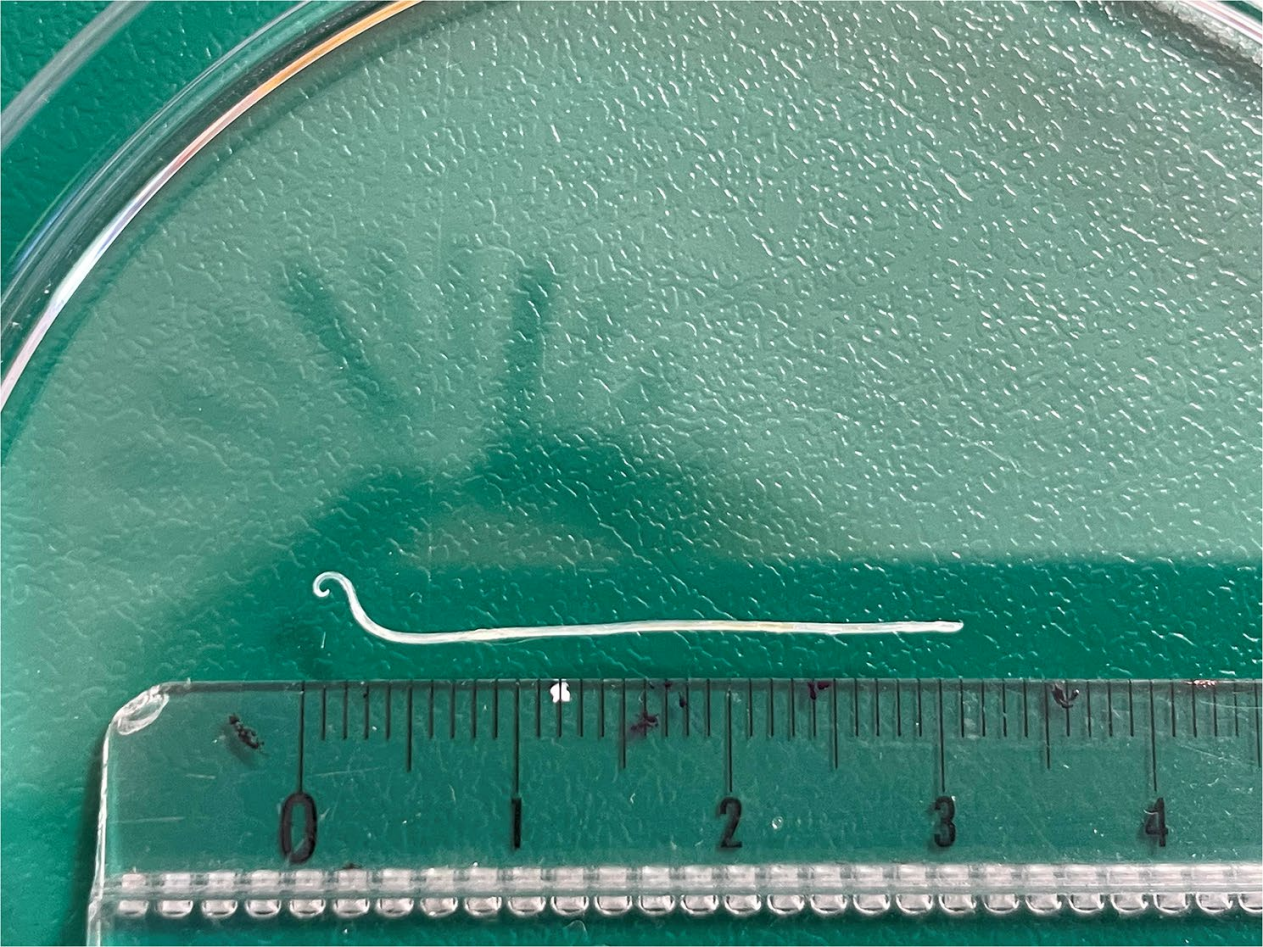
Parasitological direct examination showing a 31 mm male Loa loa with its terminal end, characterized by a crossed shape, identifying its male sex

## Electronic supplementary material

Below is the link to the electronic supplementary material.

**Supplementary Material 1**: Video in the supplementary file. The eyeworm was extracted under short general anesthesia.

## Data Availability

No datasets were generated or analysed during the current study.
